# Flexible analysis of spatial transcriptomics data (FAST): a deconvolution approach

**DOI:** 10.1186/s12859-025-06054-y

**Published:** 2025-01-31

**Authors:** Meng Zhang, Joel Parker, Lingling An, Yiwen Liu, Xiaoxiao Sun

**Affiliations:** 1 Department of Mathematics, University of Arizona, 617 N. Santa Rita Ave., Tucson, AZ 85721 USA; 2 Department of Epidemiology and Biostatistics, University of Arizona, 1295 N. Martin Ave., Tucson, AZ 85721 USA; 3 Department of Agricultural and Biosystems Engineering, University of Arizona, 1177 East Fourth Street, Tucson, AZ 85721 USA

**Keywords:** Spatial transcriptomics, Reference-free, Deconvolution, Non-negative matrix factorization

## Abstract

**Motivation:**

Spatial transcriptomics is a state-of-art technique that allows researchers to study gene expression patterns in tissues over the spatial domain. As a result of technical limitations, the majority of spatial transcriptomics techniques provide bulk data for each sequencing spot. Consequently, in order to obtain high-resolution spatial transcriptomics data, performing deconvolution becomes essential. Most existing deconvolution methods rely on reference data (e.g., single-cell data), which may not be available in real applications. Current reference-free methods encounter limitations due to their dependence on distribution assumptions, reliance on marker genes, or the absence of leveraging histology and spatial information. Consequently, there is a critical need for the development of highly flexible, robust, and user-friendly reference-free deconvolution methods capable of unifying or leveraging case-specific information in the analysis of spatial transcriptomics data.

**Results:**

We propose a novel reference-free method based on regularized non-negative matrix factorization (NMF), named Flexible Analysis of Spatial Transcriptomics (FAST), that can effectively incorporate gene expression data, spatial, and histology information into a unified deconvolution framework. Compared to existing methods, FAST imposes fewer distribution assumptions, utilizes the spatial structure information of tissues, and encourages interpretable factorization results. These features enable greater flexibility and accuracy, making FAST an effective tool for deciphering the complex cell-type composition of tissues and advancing our understanding of various biological processes and diseases. Extensive simulation studies have shown that FAST outperforms other existing reference-free methods. In real data applications, FAST is able to uncover the underlying tissue structures and identify the corresponding marker genes.

**Supplementary Information:**

The online version contains supplementary material available at 10.1186/s12859-025-06054-y.

## Introduction

Spatial transcriptomics has been rapidly expanding during the past decade [1–4]. It captures gene expression while preserving the spatial structure and information of the tissue. After sequencing, unique coordinates and gene expression levels of each spot are retained. Based on spatial transcriptomics data, we can explore the spatial patterns of expression, tissue architectures, and cell-to-cell interactions [5–9]. Several techniques of spatial transcriptomics are commonly used. For example, fluorescence imaging-based methods (e.g., merFISH) can provide high-resolution data with gene expression at the almost single-cell level in each spot [10, 11]. However, these methods can only perform sequencing with a limited number of predefined target genes. Next-generation sequencing (NGS) based spatial transcriptomics (e.g., 10X Visium) can provide whole-transcriptome sequencing but with a low-resolution (i.e. 55-100 $\mu m$) [12–14]. Throughout this paper, we focus on deconvolution for low-resolution spatial transcriptomics methods. Although deconvolution methods for bulk RNA sequencing (RNA-seq) data have been developed for decades, their generalizations to spatial transcriptomics data are limited due to the difficulties of including the spatial and histology information from the spatial transcriptomics data [15]. New methods explicitly designed for spatial transcriptomics are rapidly emerging [13, 14, 16–24]. Most of them utilize the reference data that are generated from single-cell RNA-seq (scRNA-seq) data. For example, SPOTlight (Bayes et al. 2021) combines seeded non-negative matrix factorization and non-negative least squares, and initializes its model using scRNA-seq data. It incorporates a large reference to improve stability. The cell2location method (Kleshchevnikov et al. 2022) used a hierarchical Bayesian framework for deconvolving spatial transcriptomic data, and negative binomial regression is used to estimate reference cell type signatures. GraphST (Long et al. 2023) is a deep learning method that uses a graph self-supervised contrastive learning strategy. It can jointly analyze multiple slides and capture spatial niches. These reference-based methods offer convincing deconvolution results when the prior knowledge about the reference is accurate, which requires domain knowledge and expertise in biology. Additionally, constructing a reference for deconvolution for a novel problem requires collecting and processing single-cell data when a problem-specific reference is unavailable, making it financially challenging for many labs to get accurate deconvolution results. To overcome the limitations, reference-free methods have been developed. To the best of our knowledge, only a few reference-free methods are available. For example, STdeconvolve is a reference-free spatial deconvolution method built on a latent Dirichlet allocation (LDA) model [23]. STdeconvolve achieves comparable accuracy with reference-based methods and outperforms reference-based methods when golden reference data is not available. LDA encodes the internal distributions for genes across cells and cells over spots. However, a higher drop-out or a smaller number of spots are obstacles for LDA to model such distributions, hence unable to provide highly accurate deconvolution results [23]. As spatial transcriptomics platforms approach single-cell levels, the distributional assumptions placed on the cell types within a spot, may not hold. In addition, the STdeconvolve method mainly relies on the gene expression data of each spot but ignores potential spatial dependencies within the spatial transcriptomics data. The CARD method was initially developed as a reference-based method, but it includes a built-in function CARD-free, which enables deconvolution using only marker genes of cell types [22]. CARD-free can be classified as a semi-reference-based method, because, it utilizes a limited set of marker genes as reference rather than a comprehensive reference dataset. The performance of CARD-free relies on the predefined set of cell types and their corresponding marker genes.

**Fig. 1:**
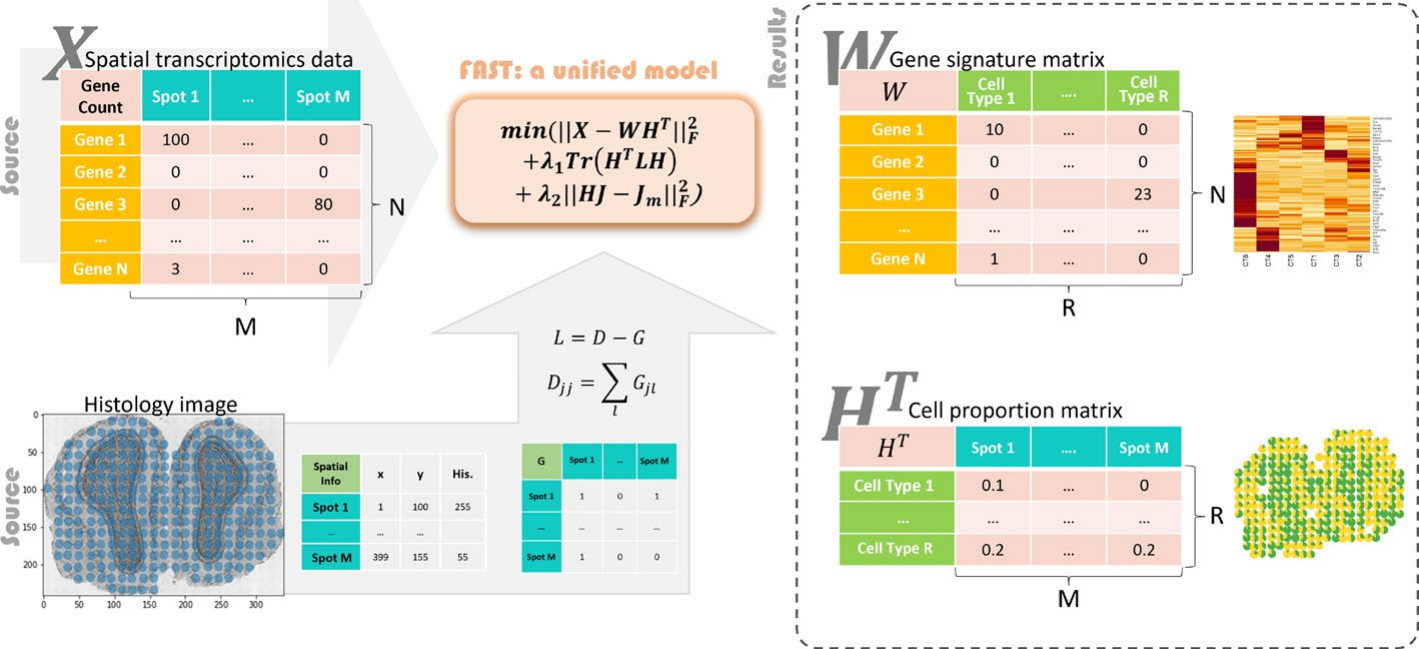
Illustration of the FAST pipeline based on regularized non-negative matrix factorization. X is an N-by-M matrix that contains gene count data per spot, while W and H are a pair of low-rank embeddings of X. W addresses the signature genes of each cell, and H contains the cell proportions in each spot. FAST, as a unified model, deconvolves X into W and H, with histology image data embedded in the objective function

In this project, we propose a novel reference-free approach called Flexible Analysis of Spatial Transcriptomics (FAST), which incorporates gene expression data, spatial data, and histology information to perform deconvolution of spatial transcriptomics data, see Fig. 1. We enhance the non-negative matrix factorization (NMF) framework by introducing two penalty terms. The first term incorporates spatial information by utilizing the graph Laplacian matrix, which is constructed by combining spatial and histology data. We introduce a straightforward method to obtain the graph Laplacian matrix in this study. Note that our method is adaptable to any graph Laplacian matrix, allowing for flexibility in its application. The second term imposes a constraint on cell proportions, encouraging their summation equals one. In summary, FAST stands out from existing methods due to its ability to impose fewer distribution assumptions, incorporate spatial tissue structures, and produce interpretable factorization results with greater flexibility. These features make FAST a versatile tool for uncovering the complex cellular composition of tissues and advancing our understanding of various biological processes and diseases that can be elucidated by spatial transcriptomics.

## Methods

FAST is a regional resolute deconvolution method that takes spatial transcriptomics data and a user-defined adjacent matrix as input to produce cell proportions of each spatial spot with the corresponding gene signature matrix as output. The gene expression matrix of spatial transcriptomics data is denoted as an N-by-M matrix $X_{N \times M}$ with N genes as rows and M spots as columns.

Consider the formulation of a simple NMF applied on spatially resolved matrix $X_{N \times M}$,

1 $$\begin{aligned} X_{N \times M}=W_{N\times R}H_{R \times M}^T+E_{N \times M}, \end{aligned}$$

where *W* is an N-by-R matrix that represents the gene signature/transcriptional profile matrix of R cell types, *H* is an M-by-R matrix that represents the abundance of R cell types in M spots, $H^T$ refers to the transpose of matrix *H*, and *E* is the error term. To minimize the error term, the objective function can be expressed as,

2 $$\begin{aligned} \left| \left| X-WH^T\right| \right| _F, \end{aligned}$$

where $\left| \left| \cdot \right| \right| _F$ is the Frobenius norm.

To incorporate the spatial information and the biological nature of the tissues into the objective function in (2), we add two regularization terms and construct the following objective function,

3 $$\begin{aligned} \left| \left| X-WH^T\right| \right| _F^2+\lambda _1Tr\left( H^TLH\right) +\lambda _2\left| \left| HJ-J_M\right| \right| _F^2 \nonumber , \\ \text {s.t. }W\ge 0 ,H \ge 0, \end{aligned}$$

where $Tr\left( H^TLH\right)$, referring to the trace of $H^TLH$, integrates the spatial information of spots with histology information, and $\left| \left| HJ-J_M\right| \right| _F^2$ imposes the summation to one penalty of cell proportion estimates for each spot [25]. Their regularized parameters $\lambda _1$ and $\lambda _2$ control the impact of each term. Particularly, the graph Laplacian matrix is defined as $L=\ D-G$ where $D_{jj}=\sum _{l} G_{jl}$, and *G* is the user-defined adjacent matrix for the nearest neighbor networks of spots. *J* is a R-by-M matrix with all elements equal to 1, and $J_M$ is an M-by-M square matrix with all elements equal to 1. The proposed method is flexible in a way that the adjacent matrix can be defined using various approaches. We propose one method in this paper, which is introduced in the next subsection. Another example of constructing the adjacent matrix is introduced in Supplementary Information. We solve *W* and *H* in (3) using the updating rules shown in Algorithm 1. Details of the derivation of updating rules can be found in Supplementary Information.

**Algorithm 1:**
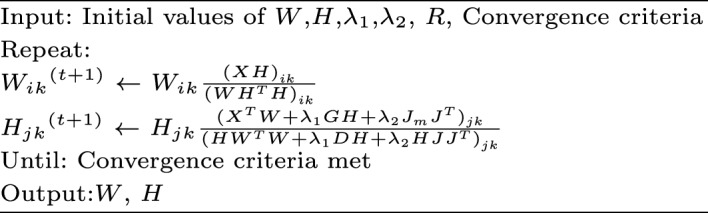
Updating rules for the FAST algorithm

The matrices *W* and *H* are initialized with random values uniformly distributed between 0 and 1. Alternatively, methods based on singular value decomposition (SVD) can also be used to initialize these matrices [26]. The rank *R*, which represents the number of cell types the algorithm will deconvolve, plays a critical role in the analysis. We provide several methods for selecting *R* in Supplementary Information.

### Construction of the adjacent matrix

The accurate construction of the adjacent matrix is critical for the success of the proposed algorithm. In this paper, we propose a straightforward method that incorporates spatial information and histology data when constructing the adjacent matrix *G*. The adjacent matrix should reflect the local spatial structures of the spots. Intuitively, spots that are physically close to each other are likely to share similar expressed gene sets and cell type distributions. However, this is not always true when organs are biologically segmented into special shapes. For example, blood vessels are tubular structures that can appear elongated or circular when viewed under a microscope. The similarity of spots in the above types of organs cannot be measured solely based on physical distance. We aim to construct an adjacency matrix based on biological proximity and physical distance. In order to find a balance between them, histology images are introduced. They are microscopic images of tissue samples on glass slides stained with various dyes to enhance the visibility of specific features, such as cell nuclei or biological features.

The proposed method calculates the adjacent matrix by integrating spatial histology and spatial coordinates in Euclidean space. We assume spots that are closer both histologically and spatially tend to have similar cell type distribution. Therefore, we compute Euclidean distances of histology and 2D coordinates of spots. The distance between two spots on histology can be calculated by measuring the difference in their median intensities over a sub-region after converting the images to grey-scale ones. In this work, the sub-region is defined as a 5-by-5 square centered around each spot, and the median intensity of the 25 spots is reserved as the value of the corresponding spot. The entries of the adjacent matrix are given by,

4 $$\begin{aligned} G_{ij}^2=\left( x_i-x_j\right) ^2+\left( y_i-y_j\right) ^2+\beta \left( z_i-z_j\right) ^2, \end{aligned}$$

where $x_i$, and $y_i$ are spatial coordinates of the *i*th spot, and $z_i$ is the gray-scaled median intensity of a spot on the histology image. The parameter $\beta$ controls the relative scale of median intensity and spatial coordinates of spots. Some histology images are vague and less informative, and $\beta$ should be assigned with a smaller number in this case. Our recommended $\beta$ is

5 $$\begin{aligned} \beta =\frac{max\left( x_i-x_j\right) ^2+max\left( y_i-y_j\right) ^2}{max\left( z_i-z_j\right) ^2}. \end{aligned}$$

We also use a sparse adjacent matrix to improve the efficiency of the proposed algorithm [25]. Particularly, we only keep the top five largest values in each row of *G*, while the rest of the values are set to zeros.

### Evaluation

Proper annotation of cell types improves the capability of biological interpretation of the results. In the simulation studies, we use a data-driven method to identify the cell type for each factor in *W* and *H*. In particular, we calculate the correlation of each factor in *W* with the true gene signature vectors of all cell types. The cell type with the highest correlation value is assigned to annotate the factor.

To evaluate the performance of the methods in the simulation studies, we utilize multiple evaluation criteria. Average Pearson correlation coefficients were computed to measure the mean correlation between the true and estimated cell proportions over all cell types. Additionally, Root-mean-square error (RMSE) was calculated to measure the differences between the estimated and true cell type proportions. If we perform downstream clustering analysis using cell proportion matrices, we will evaluate the clustering performance using the adjusted rand index (ARI) for comparison.

## Results

We conducted extensive simulation studies and real applications on three spatial transcriptomics datasets to demonstrate the performance and capability of FAST and compared its results with two reference-free methods currently available [22, 23]. The details of tuning parameter selection can be found in Supplementary Information.

### Simulation studies

**Fig. 2:**
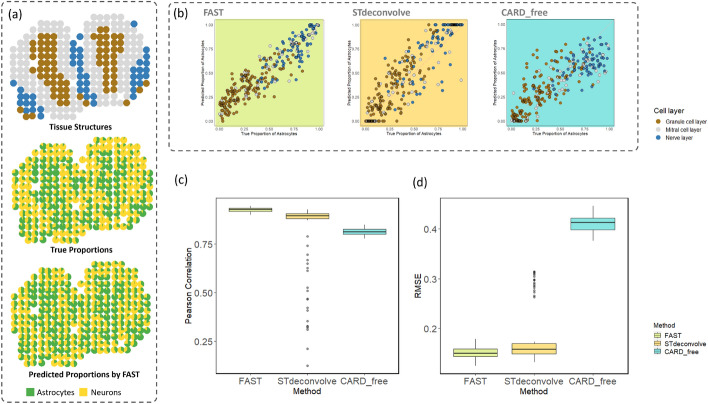
Simulation results. **a** The tissue structures, true proportion pie chart, and predicted proportion pie chart by FAST. **b** Scatter plots showing the distance between predicted and true proportions of the astrocytes for three methods. **c** Performance comparison of three methods in terms of Pearson correlation for 100 replicates. **d** Performance comparison of three methods in terms of RMSE for 100 replicates

There exist two popular simulation strategies in generating spatial transcriptomics data [21, 22]. We chose to use the simulation method based on single-cell data. Particularly, we selected cells according to a pre-defined distribution from a single-cell dataset and took the summation of the gene expression levels of the selected cells to fit each spot from the spatial transcriptomics.

**Table 1:** Simulation Settings

Region	$\hbox {Spots}^{1}$	$\hbox {Dominant}^{2}$	$\hbox {Parameters}^{3}$
Granule cell layer	75	Astrocytes	$\alpha _1=1,\alpha _2=3$
Mitral cell layer	140	Neurons	$\alpha _1=3,\alpha _2=1$
Nerve layer	45	Balanced cell type	$\alpha _1=1,\alpha _2=1$

$^{1}$ Number of spots

$^{2}$ Dominant cell type of a region

$^{3}$ Parameters of the Dirichlet distribution

The mouse olfactory bulb (MOB) is an important organization of the nervous system located at the front of the brain in mice. It receives and processes signals from olfactory receptor neurons and outputs information to other parts of the system involved in odor detection and processing. Research on MOBs helps researchers to understand the human brain structure and operation of the olfactory system to develop biomimetics smell sensors [27, 28]. The MOB spatial transcriptomics data are well-annotated, which can serve as a good reference when benchmarking MOB spatial transcriptomics analysis. MOB has a layered structure. In the simulation study, we used three layers. The olfactory nerve layer is the outermost layer which contains the axons of the olfactory receptor neurons that originate in the nasal cavity. The mitral cell layer contains mitral cells which are the key output neurons of the olfactory bulb. The granule cell layer is the innermost part and mainly contains granule cells which are inhibitory interneurons [29–31]. We used single-cell RNA-seq data with 18,215 genes and two cell types from the mouse nervous system to construct a spatial transcriptomics dataset on mouse olfactory bulbs with 260 spots [32]. Then, we selected top differentially expressed genes based on the Wilcoxon signed-rank test with an adjusted cutoff p-value $1\times {10}^{-5}$, resulting in 5,160 selected genes. A Dirichlet distribution was used to determine the proportions of each selected cell type, see Table 1. We used two cell types of astrocytes and neurons. For the 75 spots of the granule cell layer, astrocytes is the dominant cell type with $\alpha _1=1$,$\alpha _2=3$, and neurons is the dominant cell type in the 45 spots of the nerve layer. The rest 140 spots from the mitral cell layer have both cell types balanced distributed with $\alpha _1=\alpha _2=1$.

The spatially resolved pie chart in Fig. 2a shows clear patterns across the three layers of the tissue. Figure 2b shows the scatter plots of the true and calculated proportions of astrocytes across three cell layers. The closer the dots are to the 45-degree line, the better the performance. Results from FAST are consistently closer to the 45-degree line than the outputs from the other methods. This is further supported by the circular bar charts in Supplementary Information. Figure 2c, d show the results of 100 simulation replicates comparisons using Pearson Correlation and RMSE, respectively. FAST demonstrates the highest Pearson Correlation and the lowest RMSE, indicating more accurate performance compared with the other two methods. The average Pearson correlation coefficient of the proposed method was 0.93, with an increase of 0.11 compared with the best result of the other two reference-free methods. The RMSE was 0.15 on average with a corresponding improvement of 0.03. FAST also has the lowest standard deviation (i.e., 0.010 and 0.011) for both measurements, implying consistent and stable performance. More simulation results with more cell types are shown in Supplementary Information. The proposed method also outperformed the methods for comparison in this setting with more cell types.

### Real data applications

We conducted real data analysis for three datasets across two platforms. Two datasets were generated from the spatial transcriptomics platform [33]. The third dataset was generated by the 10X Visium technique with a higher spatial resolution (55 $\mu$m). During the data analysis, several clusters were identified. For simplicity, we refer to these clusters as inferred cell types (CTs). It is important to note that a CT identified by the proposed algorithm may consist of one dominant cell type along with several minor cell types.

**Fig. 3:**
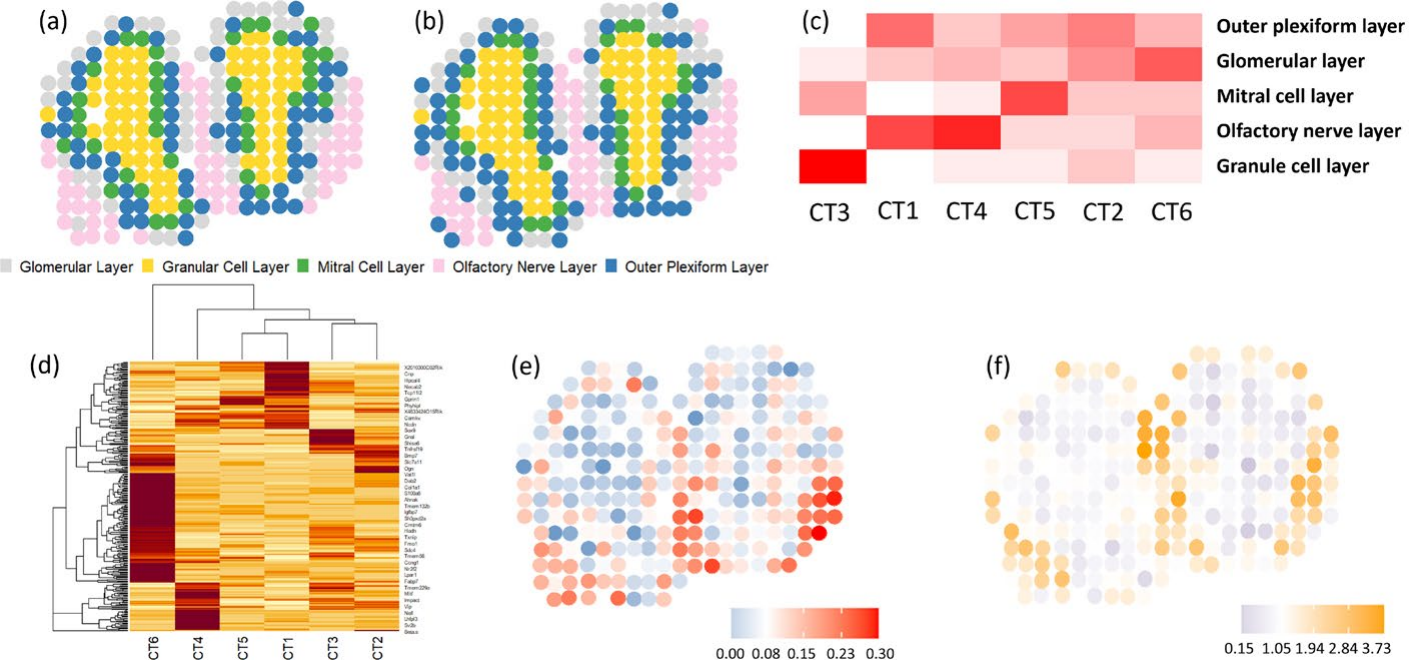
Results of FAST for the MOB data. **a** Annotated layers of MOB. This panel shows the layers that have been annotated in literature. It serves as a reference for (**b**). **b** Clustering results using the cell proportion matrix output by FAST. **c** Heatmap of the factor matrix *H*. It visualizes the distribution of cell types across the annotated tissue regions. **d** Heatmap of the gene factor matrix W. It visualizes the distribution of selected genes across the FAST inferred cell types. **e** Scatter plot of the proportions of inferred cell type 1 (CT1). **f** Spatial scatter plot of the expression levels of gene Kctd12

#### FAST recovers the structures of the mouse olfactory bulbs

**Fig. 4:**
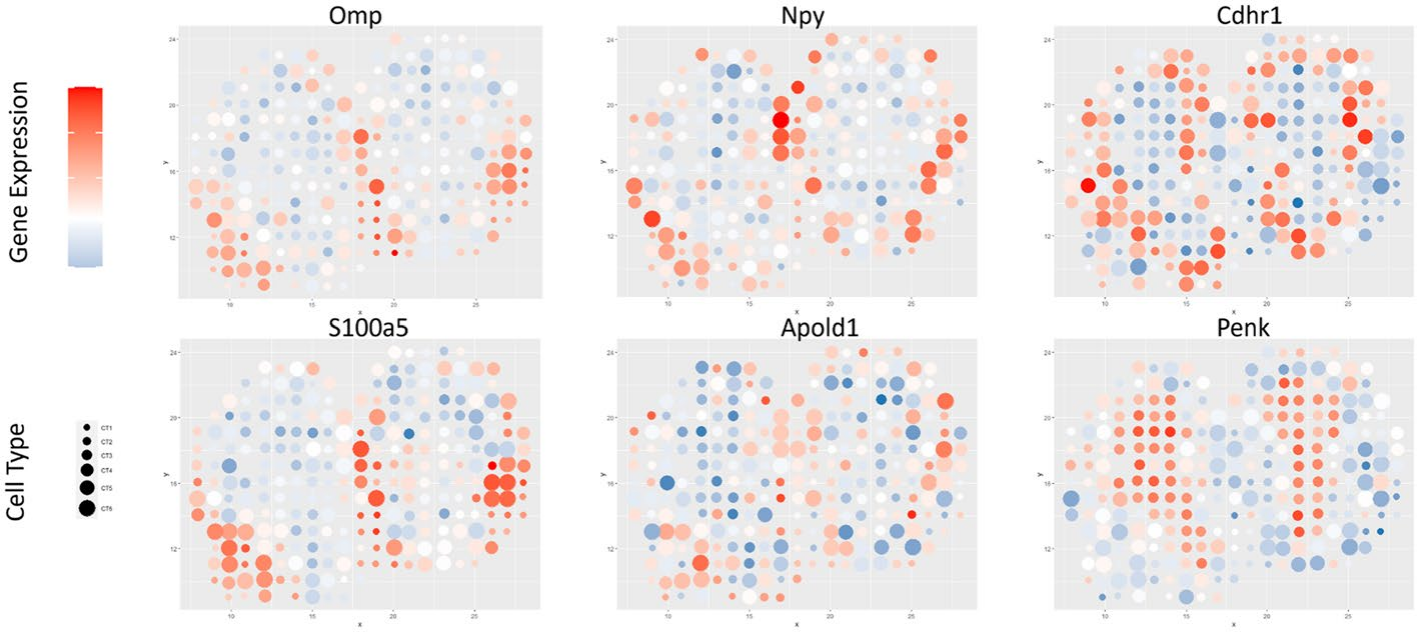
Six marker genes are shown across six factorized cell types in MOB data analysis. The color bar represents the gene expression level, and the dot size indicates the estimated proportion of each of the six cell types

Although true proportions of cell types in each spot are not available in this dataset, we can still use the annotation of MOB layers as a reliable reference for performance evaluation. There are twelve replicates for this data. Since the downstream analysis based on each replicate achieves very similar results [33], we only selected one replicate (i.e., replicate eight) for data analysis. We used a build-in function in the R package Seurat to select highly variable genes across spots [34]. Five thousand spatially variable genes were selected out of 16,218 genes. We chose the top five nearest neighbors to obtain a sparse adjacent matrix in FAST. MOB is structured in layers with discriminable cell types and functions. In this tissue slide, five layers are annotated from the outermost layer inward as the olfactory nerve layer (ONL), the glomerular layer (GL), the outer plexiform layer (EPL), the mitral cell layer (MCL) and the granule cell layer (GCL). Figure 3a shows the annotations of different layers for 260 spots. Figure 3b shows the clustering results using the K-means clustering algorithm based on cell proportion matrix *H* from the FAST algorithm. The heatmap of cell proportion matrix *H* is shown in Fig. 3c, in which different layers are well separated based on the dominant cell types. For instance, the first inferred cell type (CT1) was the dominant cell type of the olfactory nerve layer, which was illustrated in Fig. 3e. To demonstrate the capability of FAST in detecting marker genes, we generated a heatmap of gene expression profiles of all cell types, as shown in Fig. 3d. The distinct and coherent grouping of genes observed in the heatmap demonstrates the biologically interpretable results obtained from FAST. Our algorithm can also identify marker genes. In Fig. 3f, the marker gene Kctd12 of CT1 was only expressed in the spots associated with the olfactory layer [33]. The visualization provides evidence that FAST can recover the heterogeneity of tissue structures of MOB at the cell and gene expression levels. We present additional comprehensive gene and cell type coexpression plots in Fig. 4 [22, 33, 35]. Patterns of gene expressions are visualized together with the dominant cell types across spots which are represented by dot size. For example, in the last panel of Fig. 4, the heatmap visualizes the expression pattern of gene Penk which has higher gene expression levels in dominant CT3. This shows Penk serves as a marker gene for CT3.

#### FAST distinguishes cancer regions in different stages

**Fig. 5:**
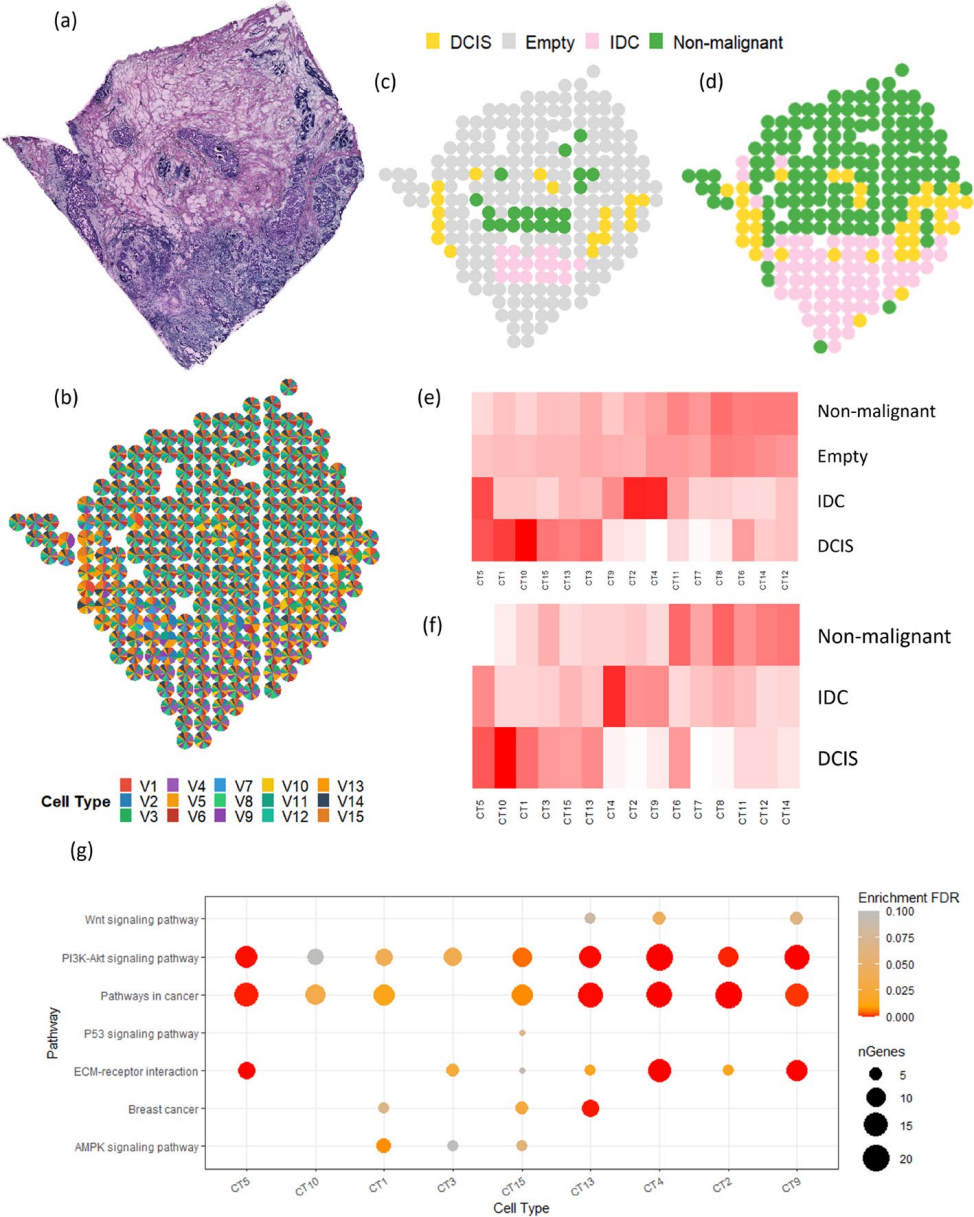
Results of FAST for the breast cancer data. **a** Histology image of the breast cancer tissue slide. It shows the original histological image of the breast cancer tissue slide, which serves as a reference for subsequent analysis **b** Cell proportion pie chart by FAST. It illustrates the proportions of the 15 cell types within the tissues at each spot inferred by FAST, providing an overview visualization of the cellular composition. **c** Annotated tissue types. This panel shows the regions that have been annotated based on known knowledge. It serves as a reference in this analysis. Grey spots remain unclassified in literature. **d** Tissue types generated by FAST. It presents the tissue types predicted using FAST. **e** Cell type by annotated tissue region heatmap. It visualizes the distribution of cell types across the annotated tissue regions. **f** Cell type by FAST tissue region heatmap. To compare with (**e**), it depicts the distribution of cell types within the tissue regions as defined by FAST. **g** Dotplot of gene enrichment results. It displays the results of gene enrichment analysis across different cell types. The horizontal axis represents the cell types identified by FAST, while the vertical axis shows the common and distinct biological pathways across IDC and DCIS regions. The color gradient of the dots corresponds to the False Discovery Rate (FDR) of the enrichment, with more significant pathways shown in a deeper color. The size of each dot indicates the number of genes (nGenes) associated with the enrichment within each pathway

The second study provides downstream analysis based on the deconvolution of human breast cancer tissues aiming to assist cancer diagnosis and treatments using spatially resolved transcriptomics data, see Fig. 5a [33]. As the most common cancer type, breast cancer has the largest incidence rate in women worldwide [36]. Identifying cellular heterogeneity greatly assists cancer diagnosis [37]. Ductal carcinoma in situ (DCIS) is a non-invasive breast cancer commonly confined to the milk ducts, and invasive ductal carcinoma (IDC) is invasive and can spread to other body parts. Distinguishing between the two types of breast cancer is critical for determining the best treatment from all the options like surgery, radiation therapy, and chemotherapy [38, 39]. In literature, partial annotation is available for DCIS, IDC, and non-malignant regions, see Fig. 5c [40]. K-means clustering based on the estimated cell proportions of FAST can recover the annotated spots and extend the annotations to those areas that were previously unclear, see Fig. 5b, d. The cell abundance analysis showed the dominant cell types in different regions, see Fig. 5e. For instance, inferred cell type 5 (CT5) was the only cell type with high abundance in both DCIS and IDC clusters. In addition, CT1 and CT10 were two of the dominant cell types in the DCIS cluster, while CT2 and CT4 were the dominant cell types of the IDC cluster. We also conducted a gene enrichment analysis on dominant cell types of tissue clusters [41, 42]. Figure 5g are pathways of the common and distinct cell compositions between the DCIS and IDC clusters. The pathways enriched in CT5 exhibit a high degree of consistency with existing literature on breast cancer pathways (e.g., ECM-receptor interaction pathway), providing further evidence of the biological relevance of this cell type in the context of breast cancer [43]. In addition, several studies have indicated a potential association between the PI3K-Akt signaling pathway and breast cancer progression. We observed a stronger activation of the PI3K-Akt signaling pathway in CT4 compared to other inferred cell types. CT4, identified as a discriminant cell type in the IDC and DCID regions by FAST, provides new evidence of the distinguishing power of this signaling pathway in breast cancer. A list of the presented pathways can be found in Supplementary Information.

**Fig. 6:**
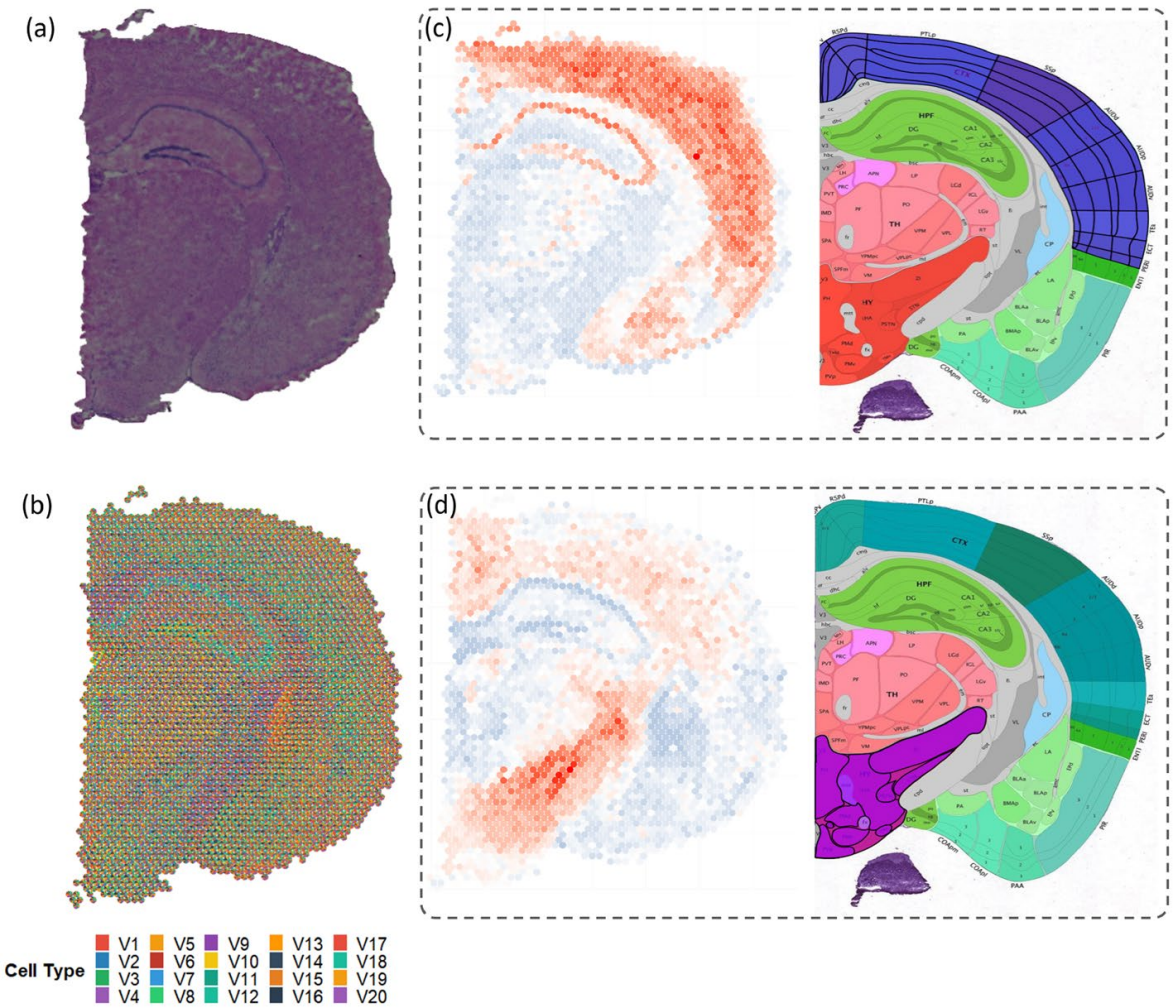
Results of FAST for the mouse brain data. **a** Histology of the mouse brain tissue slide. It shows the original histological image of the breast cancer tissue slide, which serves as a reference for subsequent analysis **b** Cell proportion pie chart by FAST. It illustrates the proportions of the 20 inferred CTs within the tissues at each spot, providing an overview visualization of the cellular composition. **c** The scatter plot of the proportion of CT2 and hypothalamus of the mouse brain. **d** The scatter plot of the proportion of CT3 and isocortex of the mouse brain

#### FAST can be applied to enhanced resolution data to recognize known brain structures

FAST can also be efficiently applied to transcriptomics data with higher spatial resolution. We analyzed transcriptomics data of a coronal section from a mouse sequenced by 10X Visium technology with 2,702 spots and 32,285 genes (Fig. 6a). We set the number of cell types to 20 and conducted deconvolution using FAST. Figure 6b is the pie chart showing the proportions of 20 cell types. To enhance the visualization of a cell distribution across all spots, we generated a proportion map for each cell type individually. This allowed us to observe the relative abundance and compare the distribution of a specific cell type with the tissue type classified by Allen Brain Atlas [44]. Figure 6c, d show the spatial distributions of CT2 and CT3, respectively, which map to the hypothalamus and isocortex of the mouse brain. Hypothalamus is located near the base of the mouse brain that is related to many physiological processes like hunger, thirst, etc. Isocortex, often referred to as neocortex, is located on the surface of the brain and controls higher cognitive functions such as perception and language.

## Discussion

In this article, we developed FAST, a novel reference-free deconvolution method for spatial transcriptomics data based on regularized NMF that integrates gene expression levels, spatial tissue structures, and histology patterns into one unified NMF model. The spatial and histology data are incorporated into the model through a graph regularization term, which utilizes a user-defined adjacent matrix. We further introduced an additional penalty on the proportion matrix to encourage the appropriate scale and uniqueness of both factorized matrices for the first time. FAST surpasses other reference-free deconvolution methods in terms of estimating cell proportions in the simulation study and showcases its potential to unlock new insights and opportunities for in-depth biological research in real data applications.

The proposed FAST algorithm is designed for the deconvolution of spatial transcriptomics data, offering a flexible framework that can produce different results based on the tuning parameter. This parameter controls the balance between the NMF reconstruction objective and the graph regularization term. Some studies have shown that when the tuning parameter exceeds 10, the results are not particularly sensitive to its exact value [25]. In this range, the regularization term encourages the factorization to adhere to the structure encoded in the similarity or adjacency matrix. In other words, the factorization prioritizes aligning the solution with the data points’ similarity structure, ensuring that neighboring points in the graph have similar representations. However, this focus on the graph structure can sometimes compromise the method’s ability to accurately reconstruct the original data matrix, as it places more emphasis on preserving the graph rather than the data itself. Conversely, when the tuning parameter is small (e.g., 0.01), the factorization more closely follows the structure of the original data and emphasizes reconstruction accuracy, resembling standard NMF. In our data analysis, we used larger values of tuning parameter (e.g., 1) to balance reconstruction accuracy with local data representation. As a result, the original NMF may perform slightly better in terms of the accuracy of reconstructing H-matrix. However, when using the estimated cell proportions for downstream analyses such as clustering, the regularized NMF tends to perform better, as it incorporates local similarity information.

Proper annotation of cell types significantly enhances the biological interpretation of the results. Generally, to assign biological labels to the inferred profiles, we implement a data-driven post-hoc annotation process based on prior knowledge, such as the dominant cell types in spatial regions or marker genes of specific cell types from single cell data. Several databases or tools are available for cell type annotation [45]. This process involves comparing the FAST-inferred profiles (*W* matrix) to known gene expression patterns from single-cell studies.

To enhance the capabilities and applicabilities of FAST, there are several directions that can be explored for future extensions and improvements. First, improving the adjacent matrix with extra information. The current adjacent matrix is calculated using spatial coordinates and the intensities of histology. A promising direction for improvement lies in defining the similarity of two spots using deep learning feature (i.e., texture) detection. Color alone is not the sole resource that can be extracted from an image, and it is vital to incorporate a comprehensive observation of histology. In addition, users have the flexibility to modify the adjacent matrix using their domain knowledge of the tissue structure and control the impact of the graph regularization term according to the level of information that the adjacent matrix contains. Second, the current updating rules are derived using the Frobenius norm in the formulations, a straightforward improvement would be to replace the Frobenious norm with Kullback–Leibler divergence and compare the performance with the current framework [46]. Last, FAST is not limited to a specific domain, and it can be applied to other deconvolution applications with minor modifications on the adjacent matrix. For example, FAST could easily be extended to any problem requiring proportional penalty. This will allow users to benefit from the improved stabilization of the NMF algorithms by inducing a sum-to-one penalty term.

## Supplementary Information

Supplementary file 1

## Data Availability

The FAST R package based on C++ is freely available on GitHub (https://github.com/shawnstat/FAST)
