# Supplementary material for: Flexible analysis of spatial transcriptomics data (FAST): a deconvolution approach
Source: BMC Bioinformatics. 2025 Jan 31;26:35. doi: 10.1186/s12859-025-06054-y (PMC11786350; doi:10.1186/s12859-025-06054-y)
Supplement: Supplementary file 1 [file 12859_2025_6054_MOESM1_ESM.pdf]

Supplementary Information for Flexible Analysis of Spatial Transcriptomics (FAST): A Deconvolution Approach

Meng Zhang[1], Yiwen Liu[2], Joel Parker[2], Lingling An[3*], and Xiaoxiao Sun[2*]

[1]Department of Epidemiology and Biostatistics, University of Arizona
[2]Department of Mathematics, University of Arizona
[3]Department of Biosystems Engineering, University of Arizona

S1 Deriving updating rules using multiplicative rule

To minimize the proposed objective function, we employ multiplicative updating rules to iteratively update the values of W and H until an optimal solution is achieved. The original algorithm proposed by Lee et al. was designed for solving the basic NMF problem, but it has since been adapted and extended for use in various NMF formulations. The Lagrange function of the formulation of FAST is derived as

$$\begin{aligned}\mathcal{L} = & \left|\left|X - WH^T\right|\right|_F^2 \\ & + 2\lambda_1 Tr\left(H^T L H\right) + \lambda_2 \left|\left|HJ - J_m\right|\right|_F^2 \\ & + Tr\left(\Psi W^T\right) + Tr\left(\Phi H^T\right),\end{aligned} \tag{S1.1}$$

where Ψ and Φ are the Lagrange multipliers for the non-negative constraints. It can be further modified for brevity and convenience as

$$\begin{aligned}\mathcal{L} = & Tr\left(XX^T\right) - Tr\left(XHW^T\right) + Tr\left(WH^T HW^T\right) \\ & + 2\lambda_1 Tr\left(H^T L H\right) + \lambda_2 Tr\left(HJJ^T H^T\right) - 2\lambda_2 Tr\left(HJJ_m^T\right) \\ & + \lambda_2 Tr\left(J_m J_m^T\right) + Tr\left(\Psi W^T\right) + Tr\left(\Phi H^T\right).\end{aligned} \tag{S1.2}$$

Taking partial derivatives of W and H, respectively, yields,

$$\begin{aligned}\frac{\partial \mathcal{L}}{\partial W} = & -2XH + 2WH^T H + \Psi, \\ \frac{\partial \mathcal{L}}{\partial H} = & -2X^T W + 2HW^T W \\ & + 2\lambda_1 LH + 2\lambda_2 HJJ^T - 2\lambda_2 J_m J^T + \Phi.\end{aligned} \tag{S1.3}$$

Set both equations equal to 0, yields,

$$\begin{aligned}-2XH + 2WH^T H + \Phi = 0 \\ -2X^T W + 2HWTW + 2\lambda_1 LH \\ +2\lambda_2 HJJ^T - 2\lambda_2 J_m J^T + \Phi = 0.\end{aligned} \tag{S1.4}$$

Apply the Karush–Kuhn–Tucker (KKT) conditions to remove Ψ and Φ elementwisely,

$$\begin{aligned}-\left(XH\right)_{ik} + \left(WH^T H\right)_{ik} = 0 \\ -\left(X^T W\right)_{jk} + \left(HWTW\right)_{jk} + \lambda_1 \left(LH\right)_{jk} \\ +\lambda_2 \left(HJJ^T\right)_{jk} - \lambda_2 \left(J_m J^T\right)_{jk} = 0,\end{aligned} \tag{S1.5}$$

where $\cdot_{ij}$ denotes the elements of the matrix [213]. Recall that $L = D - G$. The final updating rules are obtained as,

$$W_{ik}{}^{(t+1)} = W_{ik}\frac{(XH)_{ik}}{(WH^TH)_{ik}}, \tag{S1.6}$$

$$H_{jk}{}^{(t+1)} = H_{jk}\frac{(X^TW + \lambda_1 GH + \lambda_2 J_m J^T)_{jk}}{(HW^TW + \lambda_1 DH + \lambda_2 HJJ^T)_{jk}}. \tag{S1.7}$$

By iteratively updating W and H until convergence using Algorithm 1, we obtain the optimized low-dimensional representations of X.

S2 Another example to calculate adjacent matrix

Our framework is versatile, allowing for deconvolution based on various adjacency matrices. Another example to calculate the adjacent matrix relies on a learned graph from a graph convolutional network [4]. Hu et al. proposed SpaGCN to train spatial transcriptomic data with a spatial convolutional network that simultaneously considered histology, spot location, and gene expression levels. In SpaGCN, a node is a spot of gene expression data, and two spots are connected by an edge. The edge of each spot will be trained to reflect the similarity of each spot/node. The weights on each edge after training can be served as an optimal adjacent matrix reflecting the overall closeness between two spots.

S3 Rank selection

The NMF framework requires specifying the rank of the low-dimensional embedding, which corresponds to the number of cell types we aim to recover. Determining the appropriate rank can be guided by combining prior domain knowledge of tissue types with the specific research objectives. For instance, single cell data is often available for the same tissue used in spatial sequencing, allowing the rank to be set to the number of cell types present [5]. This approach is generally preferred in data analysis. However, in cases where single cell data are unavailable, data-driven methods can be employed to estimate the rank. Miller et al. have discussed data-driven methods that utilize pixel proportion to determine the appropriate rank [6]. The default value of this proportion can be estimated from factors such as model performance (perplexity) and the mean number of total cells per spot [6]. Another popular method is check the reconstruction errors (i.e., residual sum of squares (RSS)) for each rank.

Figure S1 (a) displays the scree plot using the simulation data as an example, which shows the residual sum of squares (RSS) for each rank in the NMF analysis using the default method in the R NMF package. The scree plot helps us determine the rank by identifying the point of diminishing returns or the "elbow" in the plot. In S1 (b), we present the plots from FAST, illustrating the difference in the norm as the rank increases. This plot provides additional insight into the rank selection process. In practice, we suggest starting with a lower rank and gradually increasing it, observing the changes in model performance and biological plausibility. User can also refer to the marker gene expressions and downstream clustering results to guide the selection process. By incorporating prior knowledge and employing data-driven approaches, we can effectively determine the rank within the NMF framework for cell type deconvolution, aligning it with the specific tissue layers or types of interest, and ensuring accurate and meaningful results.

The scree plot method is simple, yet it lacks robustness. A more robust alternative is the use of the gap statistic, as described in [7]. This method involves calculating the difference (gap) in reconstruction errors between two ranks. Subsequently, the null distribution of the gap statistics is constructed using the bootstrapping method. Specifically, we first identify the rank at which the gap statistic reaches its maximum. From this point, we select the smallest rank value whose gap statistic is within one standard error (SE) of this maximum. In a simulation study with a matrix of true rank four, the gap statistics calculated and presented in Figure S2 accurately estimate the rank. We have incorporated this method into the FAST software for rank selection.

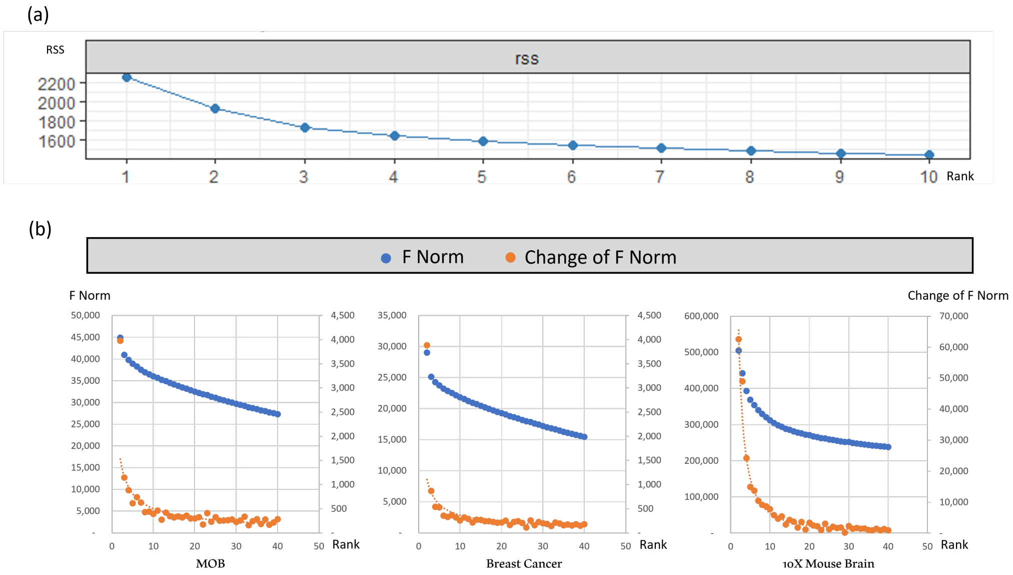

Figure S1: Performance-based plots for NMF rank selection. (a) RSS plot using the simulation data set. (b) Outputs from three real data sets.

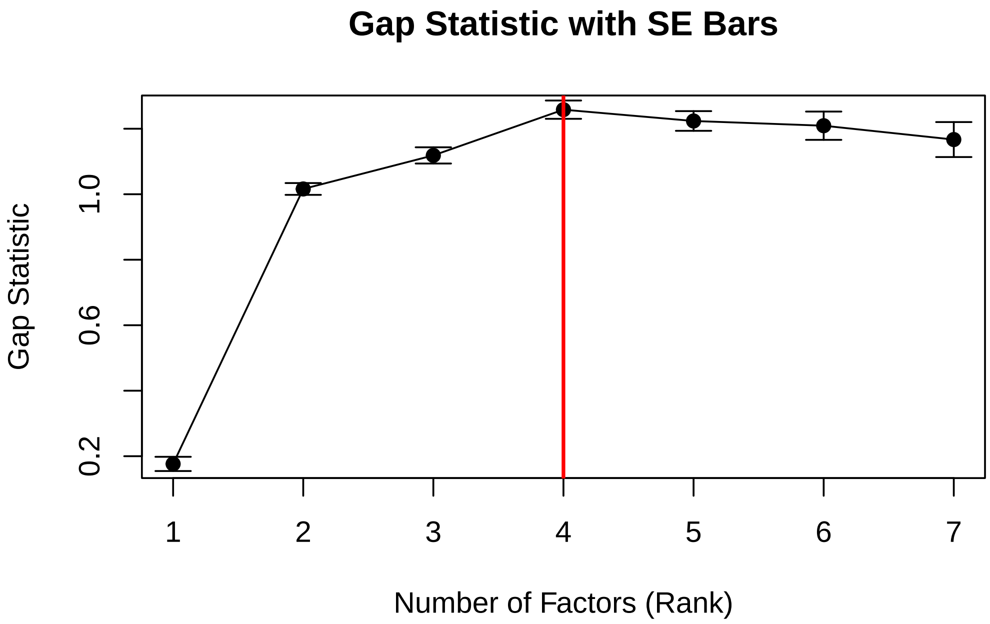

Figure S2: Gap statistics with standard error (SE bars for simulated data where the true rank is four.

S4 Parameter tuning

Figure S3 shows the performance in terms of Pearson and Spearman correlation across different choices of λ_1 using the two cell-type simulation dataset. The parameter λ_1 is not sensitive for NMF-based methods [2]. We recommended a range from [0.01,30]. In practice, λ_2 should be generally set to a value equaling to λ_1.

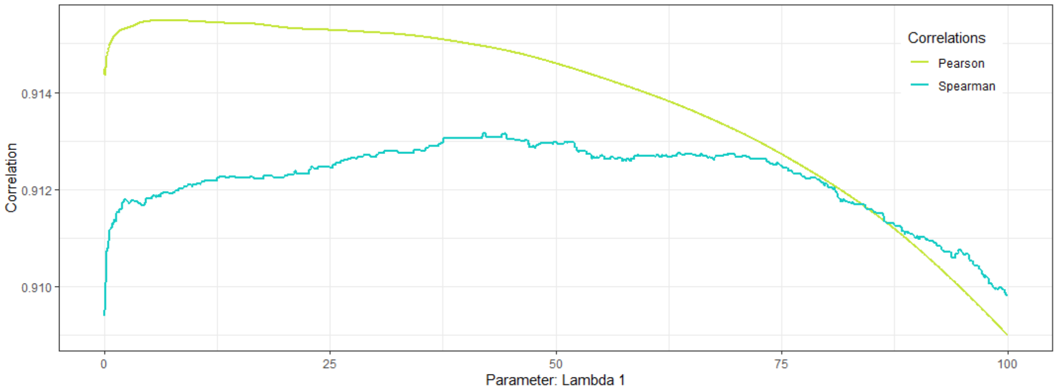

Figure S3: Performance across different choices of λ_1.

S5 Normalization

For the purpose of achieving accurate results, we standardized the gene count table using counts-per-million (CPM). The data was transformed to a logarithmic scale. A shift adjustment of 1 was introduced to this log transformation, ensuring that zero values did not pose computational issues.

S6 Polar bar chart of simulation

Polar bar charts are employed to compare the distribution of cells among three tissue types and all methods. In this chart, the axes radiate from a central point, and 260 spots are evenly arranged along a 360-degree arc. The radius of each spot represents the proportion of the corresponding cell type, ranging from 0 to 1. The plots describe the simulation setting in cell type distribution across the tissue types as well as the deconvolution results across different methods.

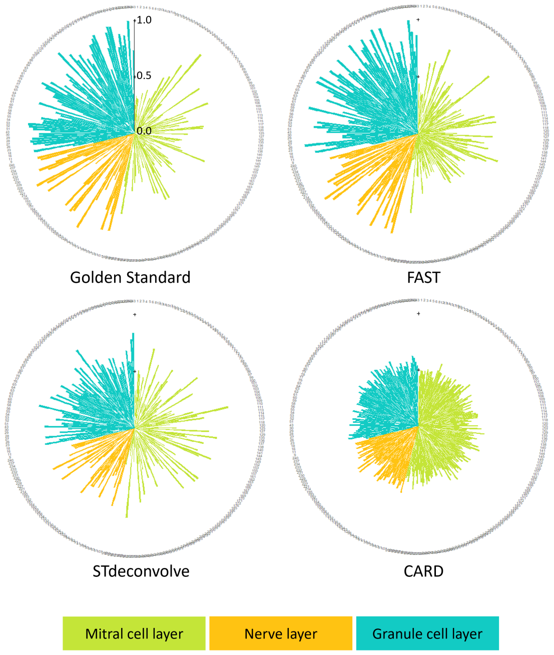

Figure S4: Polar bar chart of the proportion of Astrocytes across 260 spots.

S7 Simulation using three cell types (MOB)

We increased the cell types (Table S1) in the simulation and used Seurat to calculate the differential expression genes [1]. For each region, a dominant cell type was decided. Astrocyte was the dominant cell type for the granule layer, Neuron was the dominant cell type for the mitral layer, and Oligo was the dominant cell type for the nerve layer (Table S1). For each spot, three proportions were generated from Dirichlet distribution with concentrator parameters of all ones. The largest proportion was assigned to the decided dominant cell type of the spot, and the rest two proportions were randomly assigned to the other two cell types.

Table S1: Cell Types in Different Layers

Layers	Granule cell layer	Mitral cell layer	Never layer
Dominant Cell Type	Astrocytes	Neurons	Oligos
Total Number of Cells	2520	5851	698

Table S2: Comparison of Methods

Method	Pearson	RMSE	MAE	Run Time
FAST	0.843	0.128	0.101	2.342s
STdeconvolve	0.813	0.152	0.117	7.814s
CARD_free	0.646	0.305	0.248	3.408s

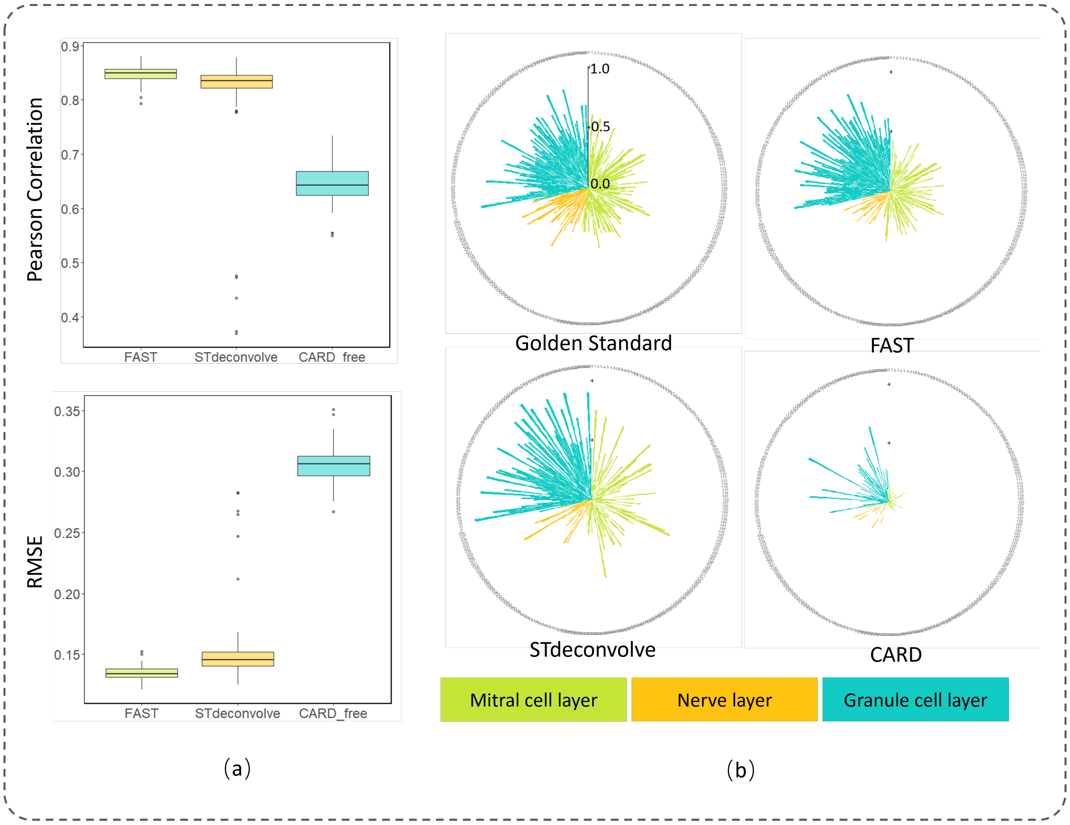

Figure S5: Three cell-type simulation results. (a) 100 replicates the performance of three methods in terms of Pearson correlation and RMSE, respectively. (b) The polar bar chart specifically focuses on Astrocytes.

With different random seeds, we ran the simulation for 100 replicates and concluded the results of all methods in Table S2 and Figure S5 (a). Table S2 summarizes the average performance matrices on 100 simulations, including Pearson correlation, RMSE, and MAE, as well as the running time on a personal PC with Intel i7-9700 CPU. STdeconvolve sees an increase in all indices when cell types increase. Figure S5 shows the polar bar charts of the Astrocytes cell proportions of the simulation ground truth and the estimated results from each method. Bars are higher in the spots of the granule cell layer (denoted as blue color) according to the simulation setting that Astrocytes is the dominant cell type of granule cell layer. All three methods have good performance in this setting. However, STdeconvolve has a tendency to amplify certain spots, resulting in an intensified pattern and increased variance. CARD underestimated Astrocytes cells across all three layers, possibly due to the choice of marker genes.

S8 Simulation with more cell types and regions using synthspot

For sufficiently large values of λ_1 (e.g., $\lambda_1 \geq 1$), the estimated cell proportions are better suited for inferring phenotypes, such as cancer subtypes, in spatial transcriptomics data. This is because the proposed framework prioritizes identifying underlying hidden structures of spots given large λ_1. To demonstrate this, we simulated spatial transcriptomics data using synthspot [3], a robust spatial transcriptomics data simulator based on single-cell data. In our simulations, we included 12 cell types and varied the number of regions from 5 to 7. The minimum number of spots allowed per region was 5. The mean and standard deviation of the normal distribution that would be used to pick the target number of counts for downsample were 30,000 and 8,000 respectively. Each region was characterized by specific dominant cell types. Effective deconvolution algorithms should demonstrate the capacity to distinctly separate these regions based on the estimated cell proportions. After obtaining the estimated cell proportions, we applied a Gaussian mixture model for clustering to obtain the predicted layer labels. To ensure a fair comparison, the number of clusters was set to match the number of regions across all methods being evaluated. The Adjusted Rand Index (ARI) was employed to assess the agreement between the predicted and the actual layer labels. As depicted in Figure S6, we evaluated the performance of three methods over 100 replications. The proposed method excelled in all scenarios involving five and seven regions. However, the performance of all methods decreased as the number of regions increased. This decline was anticipated, as clustering becomes more challenging with a greater number of clusters available.

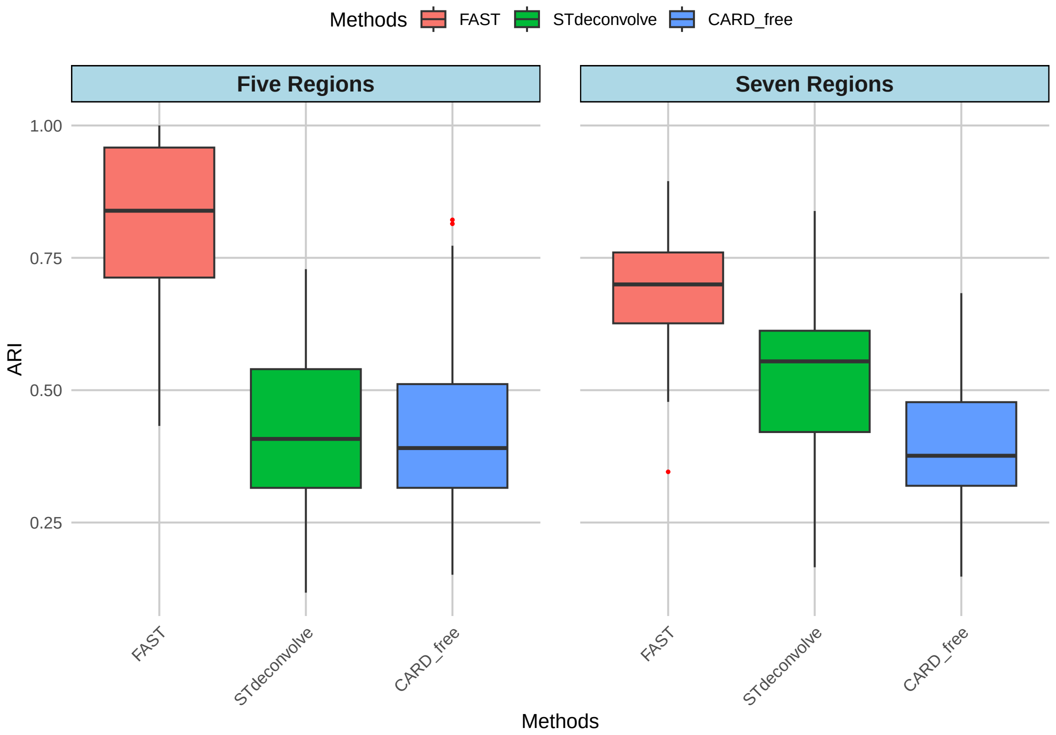

Figure S6: Performance of the methods for comparison in terms of clustering accuracy across 100 replications.

S9 Cell type distribution of 10X data

In Figure S7, we show the cell proportion distribution map of all 20 cell types derived by FAST.

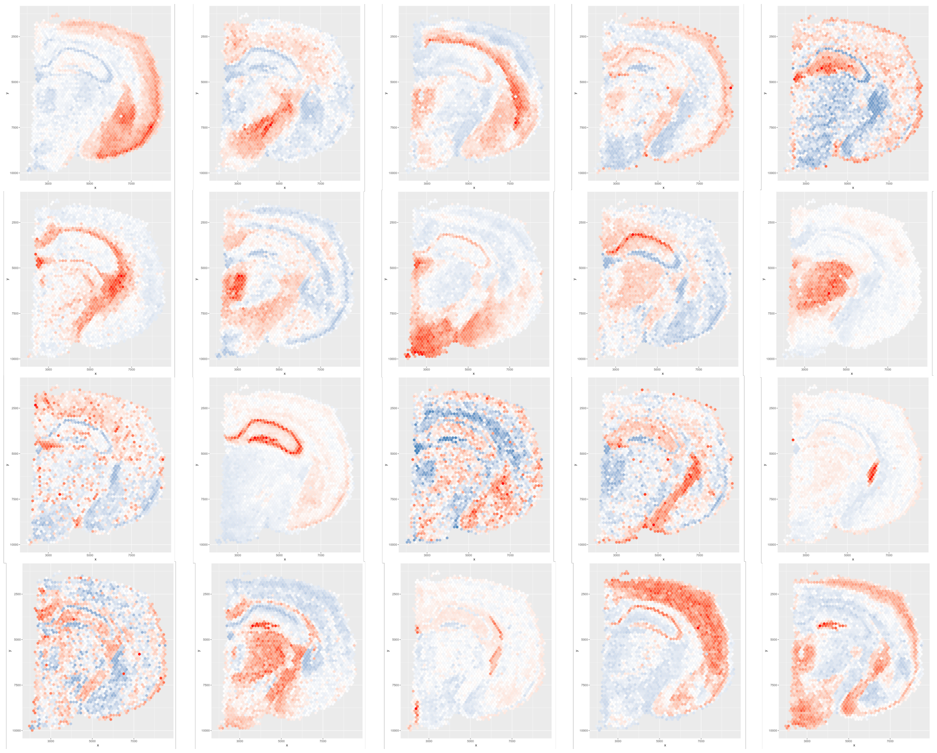

Figure S7: The cell proportion distribution map of all cell types in the 10X data.

S10 Gene enrichment analysis for the breast cancer data

Table S3 presents the cancer-related pathways and corresponding cell types. The cutoff enrichment FDR is 0.1.

Table S3: Enriched pathways detected in the breast cancer data

Pathway	CT1	CT2	CT3	CT4	CT5	CT9	CT10	CT13	CT15
AMPK signaling pathway	0.0078	-	0.0993	-	-	-	-	-	0.0581
Pathways in cancer	0.0147	2.78×10^{-7}	-	4.61×10^{-6}	0.0005	0.0013	0.0335	1.04×10^{-4}	0.0076
PI3K-Akt signaling pathway	0.0385	6.73×10^{-4}	0.0362	2.36×10^{-9}	1.81×10^{-4}	7.32×10^{-7}	0.0988	1.04×10^{-4}	0.0049
Breast cancer	0.069	-	-	-	-	-	-	3.29×10^{-4}	0.0289
ECM-receptor interaction	-	0.0159	0.0271	8.41×10^{-13}	3.70×10^{-5}	3.09×10^{-10}	-	0.0165	0.0916
Wnt signaling pathway	-	-	-	0.0423	-	0.0638	-	0.0861	-
P53 signaling pathway in cancer	-	-	-	-	-	-	-	-	0.0711

References

[1] Andrew Butler, Paul Hoffman, Peter Smibert, Efthymia Papalexi, and Rahul Satija. Integrating single-cell transcriptomic data across different conditions, technologies, and species. *Nature biotechnology*, 36(5):411–420, 2018.

[2] Deng Cai, Xiaofei He, Jiawei Han, and Thomas S Huang. Graph regularized nonnegative matrix factorization for data representation. *IEEE transactions on Pattern Analysis and Machine Intelligence*, 33(8):1548–1560, 2010.

[3] Sang-aram Chananchida, Robin Browaeys, Seurinck Ruth, and Saeys Yvan. Spotless, a reproducible pipeline for benchmarking cell type deconvolution in spatial transcriptomics. *eLife*, 12, 2024.

[4] Jian Hu, Xiangjie Li, Kyle Coleman, Amelia Schroeder, Nan Ma, David J Irwin, Edward B Lee, Russell T Shinohara, and Mingyao Li. Spagcn: Integrating gene expression, spatial location and histology to identify spatial domains and spatially variable genes by graph convolutional network. *Nature Methods*, 18(11):1342–1351, 2021.

[5] Ying Ma and Xiang Zhou. Spatially informed cell-type deconvolution for spatial transcriptomics. *Nature Biotechnology*, 40(9):1349–1359, 2022.

[6] Brendan F Miller, Feiyang Huang, Lyla Atta, Arpan Sahoo, and Jean Fan. Reference-free cell type deconvolution of multi-cellular pixel-resolution spatially resolved transcriptomics data. *Nature Communications*, 13(1):2339, 2022.

[7] Robert Tibshirani, Guenther Walther, and Trevor Hastie. Estimating the number of clusters in a data set via the gap statistic. *Journal of the Royal Statistical Society: Series B (Statistical Methodology)*, 63(2):411–423, 2001.
